# Treatment of community-onset, childhood convulsive status epilepticus: a prospective, population-based study

**DOI:** 10.1016/S1474-4422(08)70141-8

**Published:** 2008-08-01

**Authors:** Richard FM Chin, Brian GR Neville, Catherine Peckham, Angie Wade, Helen Bedford, Rod C Scott

**Affiliations:** a Neurosciences Unit, Institute of Child Health, University College London and Great Ormond Street Hospital for Children NHS Trust, London, UK; b The National Centre for Young People with Epilepsy, Surrey, UK; c MRC Centre of Epidemiology for Child Health, Institute of Child Health, University College London, UK; d Radiology and Physics Unit, Institute of Child Health, University College London, London, UK

## Abstract

**Background:**

Episodes of childhood convulsive status epilepticus (CSE) commonly start in the community. Treatment of CSE aims to minimise the length of seizures, treat the causes, and reduce adverse outcomes; however, there is a paucity of data on the treatment of childhood CSE. We report the findings from a systematic, population-based study on the treatment of community-onset childhood CSE.

**Methods:**

We collected data prospectively on children in north London, UK, who had episodes of CSE (ascertainment 62–84%). The factors associated with seizure termination after first-line and second-line therapies, episodes of CSE lasting for longer than 60 min, and respiratory depression were analysed with logistic regression. Analysis was per protocol, and adjustment was made for repeat episodes in individuals.

**Results:**

182 children of median age 3·24 years (range 0·16–15·98 years) were included in the North London Convulsive Status Epilepticus in Childhood Surveillance Study (NLSTEPSS) between May, 2002, and April, 2004. 61% (147) of 240 episodes were treated prehospital, of which 32 (22%) episodes were terminated. Analysis with multivariable models showed that treatment with intravenous lorazepam (n=107) in the accident and emergency department was associated with a 3·7 times (95% CI 1·7–7·9) greater likelihood of seizure termination than was treatment with rectal diazepam (n=80). Treatment with intravenous phenytoin (n=32) as a second-line therapy was associated with a 9 times (95% CI 3–27) greater likelihood of seizure termination than was treatment with rectal paraldehyde (n=42). No treatment prehospital (odds ratio [OR] 2·4, 95% CI 1·2–4·5) and more than two doses of benzodiazepines (OR 3·6, 1·9–6·7) were associated with episodes that lasted for more than 60 min. Treatment with more than two doses of benzodiazepines was associated with respiratory depression (OR 2·9, 1·4–6·1). Children with intermittent CSE arrived at the accident and emergency department later after seizure onset than children with continuous CSE did (median 45 min [range 11–514 min] *vs* 30 min [5–90 min]; p<0·0001, Mann-Whitney *U* test); for each minute delay from onset of CSE to arrival at the accident and emergency department there was a 5% cumulative increase in the risk of the episode lasting more than 60 min.

**Interpretation:**

These data add to the debate on optimum emergency treatment of childhood CSE and suggest that the current guidelines could be updated.

**Funding:**

An anonymous donor to UCL Institute of Child Health; the Wellcome Trust; UK Department of Health National Institute for Health Research Biomedical Research Centres Funding Scheme; Medical Research Council.

## Introduction

Convulsive status epilepticus (CSE) is the most common neurological emergency of childhood, with an incidence of between 17 and 23 per 100 000 children per year.^1^ CSE is defined as either two or more convulsions without complete recovery of consciousness between seizures (intermittent CSE) or as a single prolonged seizure that lasts at least 30 min (continuous CSE).^2^ CSE is associated with epilepsy later in life and cognitive and behavioural impairments.^3^ The treatment of CSE aims to minimise the length of seizures and treat the causes, thereby reducing adverse outcomes. Effective treatment of the seizures requires early and robust pharmacological intervention and recognition of the predictors of prolonged seizures that can be modified.

There are four phases for CSE management: prehospital; first-line treatment in the accident and emergency department; second-line treatment after the failure or absence of benzodiazepine first-line therapy; and general anaesthesia. However, there is a paucity of data on the benefits of prehospital treatment and the choice and route of administration of antiepileptic drugs (AEDs) in hospital.^4^, ^5^ Furthermore, the predictors of respiratory depression, which is an important complication of the treatment of CSE, are inadequately researched.^6^, ^7^ Neither of the current UK treatment guidelines—the Advanced Paediatric Life Support (APLS) guidelines and the National Institute for Health and Clinical Excellence (NICE) guidelines—cover the prehospital setting, despite most episodes of CSE starting in the community.^8^, ^9^ Both guidelines recommend similar hospital treatments, despite the absence of good evidence for treatments for CSE.^4^

The investigators in a prospective, population-based study of childhood CSE—the North London Convulsive Status Epilepticus in Childhood Surveillance Study (NLSTEPSS)—recruited between 62% and 84% of all potentially eligible children in north London who had CSE.^1^ The treatment given to the children in this group whose CSE began in the community was analysed. Our aims were to characterise the treatments given prehospital and in the accident and emergency department; identify the factors that are associated with seizure termination after first-line treatment in the accident and emergency department; identify the factors that are associated with seizure termination after second-line treatment in those children who failed to respond to or who had not received benzodiazepine therapy; determine which factors are associated with seizures that last for more than 60 min; and identify the predictors of respiratory depression.

## Methods

### Patients

Clinical and demographic data were collected prospectively between May 1, 2002, and April 30, 2004, on children aged between 29 days and 15 years who lived in north London and had episodes of CSE. Children who were eligible were identified through a multisource identification system that involved the clinical network that provided medical services for north London. Home postcodes were used to validate the children's residency in north London and to establish their IMD2004 (index of multiple deprivation 2004) score—a measure of the socioeconomic status for the area in which each child lived. IMD2004 scores, which are used by the UK government to direct policy and allocate resources, are the combined sums of the weighted, exponentially transformed scores of seven separate measures of deprivation (income, employment, health, education, housing, crime, and living environment). The higher the IMD2004 score, the more deprived the area is; however, because of the exponential distribution the scale is not linear, and an area with a score of 40 is not necessarily twice as deprived as an area with a score of 20.^10^

### Procedures

Data on each child who was eligible were obtained within 2 weeks of their identification, through a standardised CSE admission pro forma from copies of the accident and emergency, nursing, ambulance, and intensive care notes, and by direct interview with the attending doctors, nurses, and paramedics. The aetiology of CSE and whether there were focal features were determined, irrespective of the age of the patient, by two of the investigators (RFMC and RCS) on the basis of the history obtained from carers and by direct observation or examination by the medical team. When the assessors disagreed on the classification, conflicts were resolved by consensus or by third-party adjudication. Details of the study methods have been described previously.^1^ The study was approved by the London Multicentre Research Ethics Committee and the local research ethics committees of each health district involved. Because NLSTEPSS was a surveillance study and all data were anonymised, neither the patients nor their families provided written or verbal informed consent. There were no missing data. Treatment decisions were made by individual carers, paramedics, and the doctors who enrolled the patients, and not by the research team. Seizure termination was defined as termination of overt clinical seizure activity within 10 min of the completion of the administration of AEDs.^11^, ^12^ Seizure duration was defined as the interval between the reported onset of CSE and cessation of clinical seizure activity. Data obtained from the standardised CSE admission pro forma questionnaire included the time at which the carer first recognised seizure activity, the time at which the emergency medical services recorded being called, the time they arrived at the children, the time of their arrival at the accident and emergency department, and the time of seizure termination.

The adequacy of the doses of AEDs was defined by the doses recommended in the third edition of the APLS guideline,^13^ which was the version that was applicable during the study: 0·1 mg per kg for intravenous lorazepam; 0·5 mg per kg for rectal diazepam; 0·4 mL per kg for rectal paraldehyde; 18 mg per kg for intravenous phenytoin; and 15–20 mg per kg for intravenous phenobarbitone. Bodyweight, as estimated by staff in the accident and emergency department, was used to estimate the adequacy of doses. Doses that were lower than 80% and higher than 120% of the adequacy doses were defined as either inadequate (low) or high.^6^

### Statistical analysis

All analyses were done with SPSS version 15.0 (Chicago, IL, USA), StatXact version 4.0 (Cytel, MA, USA), or Stata SE version 9.0 (Stata, TX, USA). Logistic regression analyses were done to identify the factors that are associated with seizure termination after first-line treatment in the accident and emergency department; the factors associated with seizure termination after second-line treatment in those children who failed to respond to or who never received first-line benzodiazepine therapy; the factors that are associated with seizures that last for more than 60 min; and the factors that predict respiratory depression.

The univariate factors that were significantly associated with outcomes were identified, and whether these were independent was calculated with multivariable models. Any factors that were not significant were included in the models, to identify the factors that were associated with outcome only after adjustment for other factors. All episodes were included in the analysis, and the correlation between repeat episodes in the same child was adjusted for by the incorporation of the child as a random factor.^14^ The anthropometric factors that were assessed for all aims were age, ethnic group, sex, and IMD2004 scores. First-ever versus a history of CSE, intermittent versus continuous CSE, whether there were focal features, the aetiology of CSE, and whether the child was neurologically healthy before the onset of CSE were also recorded. With regards to treatment, we recorded whether the child had prehospital treatment or not, whether they had prehospital treatment within 15 min of the onset of CSE or not, the adequacy of dose of the first AED given prehospital, the time between onset of CSE and arrival at the accident and emergency department, the choice of AED (rectal diazepam, intravenous lorazepam, or other) on arrival at the accident and emergency department, the adequacy of the dose of the first-line AED given in the accident and emergency department, and the time between the arrival in the accident and emergency department and when the first AED was given. To identify the factors that were associated with seizure termination in the children who needed second-line treatment, the factors that were associated with seizures that lasted for more than 60 min, and the factors that predict respiratory depression, we investigated the choice of second-line AED (rectal paraldehyde, intravenous phenytoin, or other) and whether more than two doses of benzodiazepines, compared with two or fewer doses, were given.

### Role of the funding source

The funding source had no role in study design, data collection, data analysis, data interpretation, or report writing. The corresponding author had full access to all the study data and final responsibility for the decision to submit for publication.

## Results

240 episodes of CSE that started in the community were identified in 182 children (figure). Table 1 shows the characteristics of these children. Most episodes of CSE (69%) occurred in children who had pre-existing neurological abnormalities. Details of the causes of CSE in NLSTEPSS have been described previously.^1^

**Figure:**
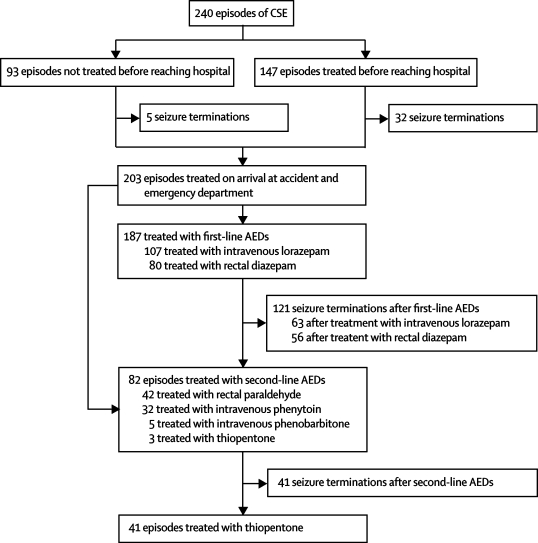
Trial profile

**Table 1:** Characteristics of episodes and children with CSE

	**Episodes (n=240)**	**Children (n=182)**
Age (years)	3·24 (0·16–15·98)	3·24 (0·16–15·98)
IMD2004 score	36·15 (6·06–70·9)	36·01 (6·06–70·90)
Time from onset to arrival at accident and emergency (min)	35 (5–514)	39 (5–514)
Duration of seizure (min)	65 (30–975)	70 (30–975)
White:non-white	95:145 (40%:60%)	65:117 (36%:64%)
Male:female	110:130 (46%:54%)	88:94 (48%:52%)
First-ever seizure:history of seizures	138:102 (58%:42%)	138:44 (76%:24%)
Intermittent seizures:continuous seizures	109:131 (45%:55%)	90:92 (49%:51%)
Focal features	84 (35%)	59 (32%)
Previously neurologically healthy	75 (31%)	71 (39%)
Prehospital treatment within 15 min of onset	68 (28%)	43 (24%)

Data are median (range) or n (%). IMD2004=index of multiple deprivation 2004.

Although not a part of the APLS guidelines, prehospital treatment was given before arrival at the accident and emergency department in 61% of episodes (figure). Of these, 93 (63%) were treated by the paramedics and 54 (37%) were treated by their carers. Rectal diazepam was the most commonly given treatment (141 episodes [96%]). The seizures were terminated in response to prehospital treatment in 37 episodes (25%), 43 (29%) episodes were treated with two or more doses of rectal diazepam, and in four of the six episodes that were treated with more than two doses of rectal diazepam the patients had respiratory depression while being transported to the accident and emergency department. Of the 141 episodes that were treated with rectal diazepam, 106 (75%) were treated with a dose that was lower than the 0·5 mg per kg recommended in the APLS guidelines for treatment on arrival at the accident and emergency department.^13^ Low-dose treatment was given by carers in 38 of 48 episodes and by paramedics in 68 of 93 episodes (79% *vs* 73%; difference 6%, 95% CI −10% to 19%). 35 of 48 children who had episodes of CSE before enrolment were given prehospital treatment by carers, whereas 71 of 93 first-ever episodes of CSE were treated, in the first instance, by paramedics. The children with first-ever CSE who were initially treated by their carers had pre-existing neurological abnormalities, including epilepsy, and had been prescribed rectal diazepam for prolonged seizures. 83 of 106 episodes that were treated with low doses had seizure activity on arrival at accident and emergency, compared with 30 of 35 episodes that were treated with the recommended doses.

203 episodes required treatment on arrival at accident and emergency, which is the starting point of the national APLS guidelines. 107 episodes (53%) were treated with a mean dose of 0·1 mg per kg intravenous lorazepam (mean difference from recommended dose 0 mg per kg [95% CI −0·01 to 0·01 mg per kg]) and 80 episodes (39%) were treated with a mean dose of 0·25 mg per kg rectal diazepam (0·26 mg per kg [0·22 to 0·30 mg per kg]), which are the first-line treatments recommended in the APLS guideline for the accident and emergency setting. The median time from arrival at accident and emergency to treatment with intravenous lorazepam was longer than the median time to treatment with rectal diazepam (7 min [1–23 min] *vs* 4 min [1–16 min]; p=0·005, Mann-Whitney *U* test). 16 episodes were not treated with the standard first-line therapy of benzodiazepines: 13 were treated with second-line therapy (nine with rectal paraldehyde, four with phenobarbitone) and three were treated with thiopentone as an anaesthetic (figure). These episodes occurred in four children with epilepsy-related CSE (one child had eight episodes, two children had two episodes each, and one child had one episode) who had episodes of CSE that did not respond to benzodiazepines.

The choice of AED and the adequacy of the dose were the only factors that were associated with seizure termination within 10 min of giving the first AED on arrival at accident and emergency as assessed with univariate analysis, and these factors were independently associated with seizure termination. In the multivariable analysis, treatment with intravenous lorazepam was more effective than was treatment with rectal diazepam, particularly if the dose was adequate (table 2).

**Table 2:** Univariable and multivariable logistic regression of seizure termination after treatment with first-line AED at accident and emergency department (n=203)

	**Univariate odds ratio (95% CI)**	**p**	**Adjusted odds ratio (95% CI)**	**p**
Rectal diazepam	1·00	..	..	..
Intravenous lorazepam	3·49 (1·53–7·99)	0·003	3·7 (1·7–7·9)	0·001
Other	1·83 (0·51–6·64)	0·36	1·5 (0·4–6·1)	0·60
Adequate *vs* low dose of first-line AED given at accident and emergency department	1·00 (1·00–1·00)	0·05	1·002 (1·000–1·003)	0·04

..=not applicable. AED=antiepileptic drug.

Of the 127 episodes that continued beyond 10 min after the first benzodiazepine was given, 107 (84%) were treated with further doses of benzodiazepines and 20 (16%) were treated with second-line treatment. A mean dose of 0·08 mg per kg intravenous lorazepam (mean difference from recommended dose 0·22 mg per kg, 95% CI 0·01–0·03 mg per kg) was the most common (n=90) second-line benzodiazepine given in hospital, and this treatment led to 17 seizure terminations. By contrast, only one of 16 episodes that were treated with a second dose of rectal diazepam (mean dose 0·21 mg per kg; mean difference from recommended dose 0·30 mg per kg, 95% CI 0·27–0·33 mg per kg) had seizure termination.

Of 82 episodes treated with a second-line AED, only 42 episodes were treated initially with the APLS-recommended AED, rectal paraldehyde (mean dose 0·28 mL per kg; mean difference from recommended dose 0·12 mL per kg, 95% CI 0·10–0·14 mL per kg). 32 of 82 episodes were treated with intravenous phenytoin (mean dose 12·4 mg per kg; mean difference from recommended dose 5·5 mg per kg, 95% CI 3·2–7·7 mg per kg). There were no statistically significant differences in the patient and seizure characteristics between groups. Two of the 42 children who were treated with paraldehyde had further clinical seizure activity within 4 h of CSE termination compared with none of the children in the other groups. No episode was treated with fosphenytoin.

Table 3 shows the factors that were identified by univariate and multivariate analyses as being significantly associated with increased seizure terminations after second-line AED and the likelihood of seizure termination after phenytoin compared with paraldehyde. No other factors were independently associated with seizure termination after the choice of initial second-line AED was taken into account.

**Table 3:** Logistic regression analyses of the factors that are associated with respiratory depression

	**Univariate odds ratio (range)**	**p**	**Adjusted odds ratio (range)**	**p**
Male *vs* female	3·3 (1·3–5·1)	0·054	..	..
First ever CSE *vs* history of CSE	2·1 (1·4–4·8)	0·055	..	..
Prehospital treatment *vs* no treatment	2·7 (1·9–5·4)	0·051	..	..
Intravenous phenytoin *vs* rectal paraldehyde as initial second-line AED	10·2 (2·4–31·2)	<0·0001	8·9 (3·0–27·0)	p<0·0001

..=not applicable. AED=antiepileptic drug.

Of 240 episodes, 44 (18%) required anaesthesia for seizure termination. Further analysis was precluded because all patients that required anaesthesia were treated with thiopentone.

The factors that were identified as significant in univariate and multivariate analyses for episodes of CSE that lasted longer than 60 min are shown in table 4. For each minute delay from the onset of CSE to arrival at the accident and emergency department there was a 5% (95% CI 3–6%) cumulative increased risk of CSE lasting longer than 60 min (p<0·0001). Thus, arrival at the accident and emergency department 40 min after the onset of CSE, irrespective of prehospital treatment, was associated with a 4·3 times greater likelihood of an episode of CSE lasting longer than 60 min compared with arrival within 10 min of onset (4·3=1·05^40−10^=1·05^30^).

**Table 4:** Logistic regression analyses of factors associated with CSE that lasted for longer than 60 min compared with CSE that lasted for 30–60 min (n=240)

	**Univariate odds ratio (95% CI)**	**p**	**Adjusted odds ratio (95% CI)**	**p**
Male *vs* female	1·8 (1·2–2·4)	0·045	..	..
No prehospital treatment *vs* prehospital treatment	3·1 (1·1–3·8)	0·001	2·4 (1·2–4·5)	0·008
Time from onset of CSE to arrival at accident and emergency department (min)	1·03 (1·02–1·08)	<0·0001	1·05 (1·03–1·06)	<0·0001
More than two doses of benzodiazepines *vs* one or two doses of benzodiazepines	3·2 (1·6–7·2)	0·0025	3·6 (1·9–6·7)	<0·0001
Intermittent *vs* continuous CSE	3·0 (1·2–5·2)	<0·0001	2·5 (1·4–4·8)	0·003

..=not applicable.

Of the 229 children treated with benzodiazepines, 120 (52%) received one or two doses and 109 (48%) received more than two doses. Children given prehospital treatment were more likely to be treated with more than two doses of benzodiazepines (81 of 147) compared with those who had their first treatment in the accident and emergency department (28 of 88 [difference 23%, 95% CI 10–35%; p=0·001]).

Episodes of intermittent CSE were 2·5 times (95% CI 1·4–4·8) more likely to last longer than 60 min compared with episodes of continuous CSE (p=0·003). To investigate whether this was related to inadequate recognition of CSE or to the management strategies, we compared the time intervals between the onset of CSE and the call to emergency medical services, the time between the call and the arrival of emergency medical services, and the time of arrival of emergency medical services and arrival at the accident and emergency department.

Children with intermittent CSE arrived at the accident and emergency department longer after the onset of CSE than the children with continuous CSE did (median 45 min [range 11–514 min] *vs* 30 min [5–90 min]; p<0·0001, Mann-Whitney *U* test). One child who arrived at accident and emergency 514 min after the onset of CSE had epilepsy and severe developmental delay and had been having intermittent seizures without recovery between convulsions, but the relevance of this was not recognised. Emergency medical services were called later for episodes of intermittent CSE than for episodes of continuous CSE (15 min [0–495 min] *vs* 5 min [1–39 min]; p<0·0001). There were no differences in emergency medical services response times (p=0·81, Mann-Whitney *U* test), but children with intermittent CSE arrived at the accident and emergency departments later than those with continuous CSE did (median time 20 min [range 2–100 min] *vs* 15·5 min [5–43 min]; p=0·037, Mann-Whitney *U* test). Similar proportions of children with intermittent CSE received prehospital treatment (95 of 187 [51%]) compared with children with continuous CSE (96 of 187 [49%]), and there was no difference between the groups in the speed of giving prehospital treatment.

Respiratory depression, which is defined as a fall in oxygen saturation below 92% in room air and a decrease in respiratory effort that requires assisted breathing at any point from the onset of CSE to seizure termination, was identified in 41 episodes (17%). Treatment with more than two doses of benzodiazepines and increasing socioeconomic deprivation were significantly associated on univariate analysis with respiratory depression (table 5). Each unit increase in the IMD2004 score has a 3% increased risk of CSE: a 30 point difference in IMD2004 scores would increase the risk of CSE 2·4 fold (1·03^30^).

**Table 5:** Logistic regression analyses of the factors that are associated with respiratory depression

	**Univariate odds ratio (95% CI)**	**p**	**Adjusted odds ratio (95% CI)**	**p**
More than two doses of benzodiazepines *vs* two or fewer doses of benzodiazepines	3·2 (1·3–7·4)	0·031	2·9 (1·4–6·1)	0·02
IMD2004	1·01 (1·00–1·04)	0·024	1·03 (1·00–1·05)	0·03

IMD2004=index of multiple deprivation 2004.

## Discussion

The results of this study provide systematic population-based data on the treatment of community-onset childhood CSE. These data add to the clinical debate on the appropriateness of current pharmacological interventions to treat potentially modifiable risk factors for seizures lasting longer than 60 min and for respiratory depression. Although there is a risk of reporting bias, particularly with regard to the start and end times of the episodes of CSE, this was minimised through our design of a population-based study with high ascertainment, prospective and early collection of data, the use of a standardised admission pro forma and copies of medical reports that were anonymous, direct interviews with medical staff, and construction of individual timelines as recorded by the emergency medical services and the accident and emergency department staff.

Treatment guidelines for CSE have previously been directed to the treatment of CSE in the hospital; however, because most childhood seizures start in the community, prehospital treatment should have an important role. Prehospital treatment reduced seizure duration in hospital-based studies in children and adults,^15^, ^16^ and our data provide evidence that prehospital treatment in children who have seizures that last for at least 30 min might also reduce seizure length. However, prehospital treatment given by emergency medical service personal is not a universal practice, and even when prehospital treatment is included in emergency treatment guidelines it is not always given.^6^ In north London, the London Ambulance Service has a policy of giving rectal diazepam to children with seizures, although this is not included in the UK APLS guidelines.^17^ Despite this treatment, a third of the children in our cohort did not receive prehospital treatment. Because most children had first-ever CSE and were previously neurologically healthy, prehospital treatment could only have been given by the emergency medical services. Therefore, giving benzodiazepines in the community to children with seizures might reduce seizure length and adverse outcomes. Although rectal diazepam was the AED used most commonly in our study, data suggest that buccal midazolam might become the preferred treatment in this setting.^18^, ^19^, ^20^, ^21^

In our study, intermittent CSE was as common as continuous CSE was but not as well recognised or managed: carers delayed seeking help, arrival at accident and emergency departments was later, and a greater proportion of episodes lasted for more than 60 min. An emergency medical services protocol that advises against the treatment of seizures that have “stopped” might contribute to under-recognition of intermittent CSE. Because responses to prehospital treatment cannot be predicted, early transport to hospital is paramount; the longer a seizure lasts, the harder it is to stop.^22^, ^23^ Early transport to hospital enables inadequate prehospital doses to be topped up, quicker first-line treatment to be given to children who have not been treated, and the use of other AEDs in those children with seizures that are refractory to benzodiazepines.

We only studied children who were taken to an accident and emergency department after having a seizure or series of seizures and did not recover consciousness for at least 30 min. Therefore, children with seizures who were successfully treated by their carers or paramedics and who did not need to attend hospital were excluded, which could explain the substantially lower response rate to rectal doses of diazepam compared with the response rates reported elsewhere.^24^

A Cochrane Review of the treatment of childhood CSE found only one eligible study,^4^ in which the authors compared intravenous diazepam with intravenous lorazepam as the first-line treatment in hospital^25^ and suggested that these drugs were similar in efficacy. However, no data to compare rectal diazepam with intravenous lorazepam (the drugs recommended by APLS and NICE) are reported. Our data suggest that intravenous lorazepam is associated with a greater likelihood of seizure termination than rectal diazepam is, without increased risk of respiratory depression. The onset of CSE is not always witnessed, and there is evidence of ongoing electrical seizure activity seen on EEG in a few patients after all clinical seizure activity has stopped, although such activity is unlikely if there is evidence of quick recovery.^25^ EEG monitoring is not widely available in the hospitals that participated in this study; therefore, we had to rely on the clinical expertise of the attending doctors. Patients in whom overt seizure activity stopped and who showed clinical evidence of recovery shortly afterwards were deemed to have had seizure termination. Continued seizure activity was presumed if they did not show evidence of recovery and they were managed accordingly. Our approach, which established the start and end times of seizure activity in CSE, is similar to the one used in other studies,^15^, ^16^, ^26^ thereby increasing the general applicability of our results.

The authors of studies in young children suggest that a single rectal dose of 0·5 mg per kg of diazepam or a single intravenous dose of 0·1 mg per kg of lorazepam can provide levels of either AED that are effective to stop convulsions within 3 min of them being given, but there is substantial variation in absorption by the rectal route.^27^, ^28^, ^29^, ^30^ In this study, children who were treated with intravenous lorazepam were treated later after onset of CSE and on arrival at hospital than children who were treated with rectal diazepam, but they still had a greater likelihood of seizure termination. Taken together, these data suggest that the speed of action of AEDs alone is unlikely to explain the observed differences between intravenous and rectal treatment.

We have shown that intravenous lorazepam can be given quickly to children with CSE, although it took 3 min longer to give than rectal diazepam did; however, this should not mean that intravenous lorazepam is slower to reach the brain. The rate of absorption of diazepam through the rectal mucosa is such that the speed of the delivery of lorazepam and diazepam to the brain are likely to be similar.^31^

We found that similar proportions of patients whose episodes were treated with adequate doses of rectal diazepam before they arrived at hospital continued to have seizure activity on arrival at accident and emergency, without an increased risk of respiratory depression, as those patients who were given inadequate doses. In hospital, giving an adequate dose of AED was independently associated with an increased likelihood of seizure termination; therefore, if rectal diazepam has to be used, we recommend that it be given at an adequate dose.

The APLS guideline recommends that a second dose of benzodiazepine is given in the accident and emergency setting. However, only 20% of children in our cohort had seizure termination after a second dose, which calls this strategy into question. Furthermore, giving a second dose might result in excessive benzodiazepine load and associated respiratory depression, particularly when the patient has had prehospital treatment. Previously reported respiratory depression after more than two doses of benzodiazepine^6^, ^32^ was confirmed in this study, irrespective of the adequacy of the initial dose. The finding that worse socioeconomic status contributes to respiratory depression highlights a potential vulnerability for ill health.^33^ The mechanism through which this association might occur is unclear. We hypothesise that it might be related to the reduced ability of parents to recognise and treat sickness and the decreased respiratory reserve in sick children; socioeconomic deprivation is associated with increased difficulties in parenting^31^ and childhood respiratory problems.^34^, ^35^ IMD2004 score increases with socioeconomic deprivation (worsening socioeconomic status).^1^ The restricted effectiveness of the second dose of benzodiazepine given in accident and emergency might be related to benzodiazepine receptor resistance, which increases with seizure length.^36^, ^37^, ^38^ These data favour the restriction of benzodiazepine treatment to two doses, irrespective of where the first dose was administered.

The APLS guidelines recommend that rectal paraldehyde is used after benzodiazepine therapy has failed. However, our results show that current practice differs: almost a half of the children received phenytoin. The absence of fosphenytoin in the treatment of CSE might be related to its exclusion from the APLS and NICE guidelines.^8^, ^9^ The only predictor of seizure termination after second-line therapy was the choice of antiepileptic drug: children who received phenytoin were about nine times more likely to have seizure termination within 10 min of treatment than the children who were treated with rectal paraldehyde were. Therefore, the treatment of children with CSE with rectal paraldehyde seems unjustified, particularly if intravenous access has been secured.

Because our study was observational with no prespecified plan for management of patients, our findings—which would support changes in the guidelines for pharmacological interventions and highlight the importance of early, aggressive treatment of CSE—should be confirmed with controlled trials. However, the recruitment of sufficient numbers of patients could be problematic, there would be substantial cost implications, and there are potential ethical difficulties with such studies. Owing to the clinical and educational implications of our study, an update of the current guidelines for the treatment of childhood CSE that incorporates a Cochrane Review of the topic would be timely.
